# Ureteral Preservation Using Fluorescent Ureteral Catheter during Laparoscopic Resection of an Appendiceal Inflammatory Pseudotumor: A Case Report

**DOI:** 10.70352/scrj.cr.25-0722

**Published:** 2026-04-22

**Authors:** Yudai Yagihashi, Ken Imaizumi, Nobuki Ichikawa, Tadashi Yoshida, Yosuke Ohno, Kengo Shibata, Chihiro Ishizuka, Noriyuki Otsuka, Shinya Tanaka, Akinobu Taketomi

**Affiliations:** 1 Department of Gastroenterological Surgery 1, Graduate School of Medicine, Hokkaido University, Sapporo, Hokkaido, Japan; 2 Department of Surgical Pathology, Hokkaido University Hospital, Sapporo, Hokkaido, Japan

**Keywords:** appendiceal inflammatory pseudotumor, fluorescent ureteral catheter, ureteral injury

## Abstract

**INTRODUCTION:**

When a bulky intra-abdominal tumor cannot be clearly distinguished from a malignancy, careful surgical planning is required to preserve adjacent organs.

**CASE PRESENTATION:**

A 31-year-old female presented to our hospital with lower abdominal pain. Contrast-enhanced CT revealed a 67-mm mass in the cecum. Colonoscopy revealed a raised lesion; however, biopsy results were inconclusive. Laparoscopic ileocecal resection with D2 lymphadenectomy was performed because of a suspected malignancy. Given the proximity of the tumor to the right ureter on preoperative imaging, a fluorescent ureteral catheter (FUC) was inserted preoperatively. Intraoperatively, near-infrared light enabled a clear ureteral visualization, thereby facilitating safe dissection and successful preservation. Her postoperative course was uneventful. Pathological examination revealed a spindle cell lesion with suppurative inflammation, and immunostaining indicated myofibroblastic differentiation consistent with an appendiceal inflammatory pseudotumor.

**CONCLUSIONS:**

This case highlights the utility of FUC-guided navigation for ureteral preservation during minimally invasive surgery for bulky intra-abdominal masses.

## Abbreviations

ALK
: anaplastic lymphoma kinase
FUC
: fluorescent ureteral catheter
IPT
: inflammatory pseudotumor

## INTRODUCTION

IPTs are benign, localized granulomatous lesions commonly found in the lungs and liver; however, their occurrence in the lower gastrointestinal tract is rare.^1)^ These lesions often resemble malignant tumors, making preoperative diagnosis and surgical planning particularly challenging.^2)^ In such cases, careful consideration is required to determine whether adjacent organs can be preserved. Herein, we report a case of an appendiceal IPT that was surgically resected laparoscopically with successful ureteral preservation using a FUC for intraoperative navigation.

## CASE PRESENTATION

**Fig. 1:**
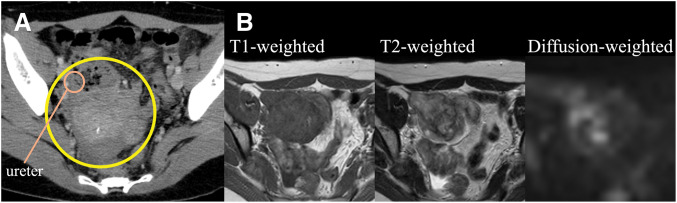
Preoperative imaging. (**A**) Contrast-enhanced CT revealed a 67-mm mass in the cecum with irregular wall thickening, heterogeneous enhancement, and encapsulated fluid collection (yellow circle). A linear high-density area within the mass suggested the presence of an appendiceal fecalith. The right ureter was closely adjacent to the mass (orange circle). (**B**) MRI revealed cecal wall thickening that appeared hypointense on T1-weighted images (left image), slightly hyperintense on T2-weighted images (center image), and partially hyperintense on diffusion-weighted images (right image).

**Fig. 2:**
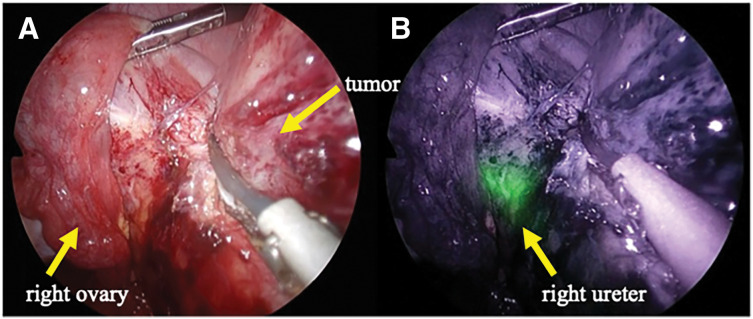
Intraoperative findings. (**A**) Intraoperatively, the tumor was located in the ileocecal region, and it was difficult to identify the anatomical relationship between the tumor and the right ureter under the white light view. (**B**) Near-infrared fluorescence imaging using VISERA ELITE II (Olympus, Tokyo, Japan) enabled easy identification of the right ureter (green color) during surgery. This allowed dissection to proceed safely along the retroperitoneum and dorsal aspect of the tumor while continuously confirming the course of the ureter.

**Fig. 3:**
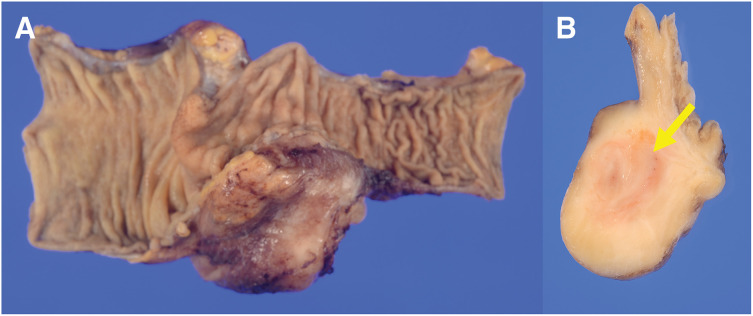
Gross examination. (**A**) The resected specimen revealed a 9.0 × 8.0 × 5.5-cm mass. A narrow tubular structure, presumably the appendix, was embedded within the mass. (**B**) The cut surface was predominantly grayish-white, with focal areas of pale yellow to orange discoloration. The narrow tubular structure is indicated by the yellow arrow.

**Fig. 4:**
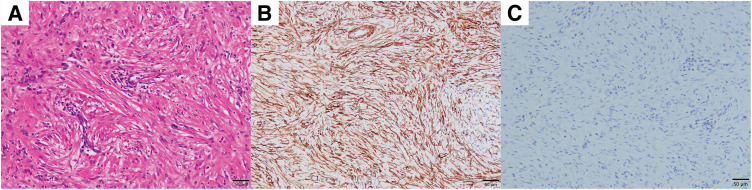
Histopathological findings. (**A**) Hematoxylin and eosin staining showed that the tumor consisted of spindle-shaped cells and central areas of suppurative inflammation. (**B**) Immunohistochemical staining showed α-smooth muscle actin positivity. (**C**) Immunohistochemical staining showed ALK negativity. ALK, anaplastic lymphoma kinase

A 31-year-old female presented to a previous hospital with lower abdominal pain. Imaging revealed an intra-abdominal mass, and the patient was referred to our hospital for further evaluation and treatment. The patient had no significant medical history. Her BMI was 21.6 kg/m^2^, and abdominal examination was unremarkable. Laboratory findings included a white blood cell count of 9800/μL and a C-reactive protein level of 1.37 mg/dL. Tumor marker levels were within normal limits. Contrast-enhanced CT revealed a 67-mm mass in the cecum with irregular wall thickening, heterogeneous enhancement, and encapsulated fluid collection. A linear high-density area within the mass suggested an appendiceal fecalith. Slightly enlarged lymph nodes were observed along the ileocolic vessels, and a small amount of ascites was present in the pelvis (**Fig. 1A**). Abdominal ultrasonography revealed a well-defined, irregular, and hypoechoic mass with internal vascularity. MRI revealed cecal wall thickening that appeared hypointense on T1-weighted images, slightly hyperintense on T2-weighted images, and partially hyperintense on diffusion-weighted images (**Fig. 1B**). Colonoscopy revealed a 30-mm elevated lesion with elastic hardness and indistinct borders in the cecum. The mucosal surface was smooth with no signs of ulceration. Biopsy specimens showed dense infiltration of inflammatory cells in the lamina propria without cytologic atypia. Immunohistochemistry revealed the possibility of a lymphoma. Differential diagnoses included cecal or appendiceal carcinoma, malignant lymphoma, and actinomycosis. Because malignancy could not be ruled out, surgical resection was performed. A laparoscopic ileocecal resection with D2 lymphadenectomy was performed. Preoperative imaging demonstrated that the tumor was in close proximity to the right ureter. Given the large size of the tumor and the anticipated difficulty in identifying the ureter during laparoscopic dissection, a highly visible FUC was selected to minimize the risk of ureteral injury. Accordingly, after induction of general anesthesia, a pig-tailed near-infrared ray catheter (NIRC; Cardinal Health, Tokyo, Japan) was inserted by urologists. A conventional 5-port laparoscopic approach was used for the procedure. The laparoscopic camera system used was a VISERA ELITE II system (Olympus, Tokyo, Japan). Intraoperatively, the tumor was located in the ileocecal region with a smooth surface and clearly demarcated border. A small amount of serous ascites was observed in the pelvis; however, no evidence of peritoneal dissemination was noted. Because of marked tissue sclerosis caused by chronic inflammation surrounding the tumor, the right ureter was difficult to identify under conventional white-light imaging, and the usual dissection plane between the dorsal aspect of the tumor and the retroperitoneum was obscured. In contrast, near-infrared fluorescence imaging enabled clear identification of the right ureter, allowing safe dissection along the retroperitoneum and the dorsal aspect of the tumor while continuously confirming the ureteral course (**Fig. 2**). This facilitated precise laparoscopic sharp dissection and enabled complete tumor resection without disruption or rupture. After ligation and division of the ileocolic vessels, the specimen was extracted intact, and intestinal reconstruction was performed using a functional end-to-end anastomosis. Operative time was 133 min with no blood loss. The ureteral catheter was removed before recovery from anesthesia. Her postoperative course was uneventful, and the patient was discharged on POD 8. Gross examination of the resected specimen revealed a 9.0 × 8.0 × 5.5-cm mass. A narrow tubular structure, presumably the appendix, was embedded within the mass (**Fig. 3A**). The cut surface was predominantly grayish-white, with focal areas of pale yellow to orange discoloration (**Fig. 3B**). Histologically, the tumor consisted of spindle-shaped cells with central areas of suppurative inflammation (**Fig. 4A**). Immunohistochemical staining showed α-smooth muscle actin positivity (**Fig. 4B**) and ALK negativity (**Fig. 4C**), suggesting myofibroblast differentiation. The final diagnosis was appendiceal IPT. All resection margins were macroscopically and microscopically negative, confirming complete resection.

## DISCUSSION

This case involved a bulky appendiceal lesion that mimicked a malignancy and required careful surgical planning to preserve adjacent organs. Laparoscopic resection with intraoperative near-infrared navigation enabled safe dissection and ureteral preservation.

Differential diagnoses for mass-forming appendiceal lesions include acute or chronic appendicitis, carcinoid tumors, mucoceles, appendiceal carcinoma, and, rarely, IPT. IPTs are benign neoplasms characterized by spindle cell proliferation and dense inflammatory infiltrates.^3)^ Polypoid lesions are classified as inflammatory fibroid polyps,^4)^ while those that form mass lesions are termed IPTs.^3)^ Among IPTs, those composed of fibroblast-like spindle cells with myofibroblastic features are referred to as inflammatory myofibroblastic tumors, which often exhibit ALK positivity.^5)^ In the present case, ALK was negative, making a diagnosis of inflammatory myofibroblastic tumor unlikely. Although IPTs frequently develop in the lungs and liver, they rarely occur in the gastrointestinal tract.^1)^ Colonic involvement is infrequent, but tends to occur in the right colon.^6)^ Although rare, reports of the coexistence of appendicitis and cecal IPT have been published. The etiology of IPT is unclear; however, it may involve immune dysregulation in response to non-infectious stimuli, such as Crohn’s disease or chronic diverticulitis.^7)^ Abundant lymphoid tissue in the appendix may promote persistent inflammation and fibroblast proliferation during tissue repair, ultimately forming a mass. Owing to nonspecific imaging findings, preoperative diagnosis is difficult, and surgical resection is commonly required.^6)^ In some reports, inflammatory appendiceal masses were suspected to be malignant, leading to combined resection of adjacent organs.^2)^ Organ preservation should be carefully considered for bulky tumors.

Ureteral injury is a serious intraoperative complication that can prolong surgery, cause urologic morbidity, and impair QOL. Its incidence is higher in laparoscopic colorectal surgery than that in open colorectal surgery.^8)^ The risk increases in high-risk situations, including those with large tumors and locally advanced cancers with invasion, severe inflammation, and dense adhesions.^8)^ Prophylactic ureteral catheterization has been used in such cases; however, conventional catheters offer limited value in laparoscopic surgery because of the lack of tactile feedback. Advanced catheter technologies have been developed to improve intraoperative visualization. Light-emitting ureteral catheters, introduced around 2010, require specialized light sources and raise concerns regarding their thermal effects on surrounding tissues.^9)^ Around 2020, FUCs emerged as a novel alternative for intraoperative ureteral visualization. These catheters utilize near-infrared fluorescence and do not emit heat, thereby minimizing the risk of thermal influence.^10)^ Although their clinical application remains limited, several reports have demonstrated the utility of FUCs in challenging colorectal procedures.^11,12)^ In particular, the use of FUCs in patients with T4 colorectal cancer has been shown to reduce conversion to open surgery and the incidence of ureteral injury, supporting their role in high-risk surgical settings.^12)^ To our knowledge, however, no prior reports have described the use of FUCs in the management of large intra-abdominal tumors of uncertain malignant potential, such as bulky appendiceal lesions mimicking malignancy. In the present case, tumor bulk accompanied by chronic inflammatory changes obscured the normal dissection plane and made ureteral identification difficult under conventional white-light imaging. The use of FUC enabled clear and continuous visualization of the ureter, facilitating safe dissection, preservation of the ureter, and complete tumor resection with secure surgical margins. This case demonstrates that FUCs can contribute not only to ureteral preservation but also to the achievement of oncologically sound surgical manipulation in tumors that are difficult to distinguish from malignancy preoperatively. Despite these advantages, the use of FUCs is associated with higher costs than conventional ureteral stents, which may limit their widespread adoption. Therefore, careful patient selection is essential, and the use of FUCs should be considered primarily in cases with a high risk of ureteral injury.

## CONCLUSIONS

FUC-guided navigation surgery is a valuable option for preserving the ureter during laparoscopic procedures for bulky intra-abdominal masses in which malignancy cannot be excluded.

## DECLARATIONS

### Availability of data and materials

Data supporting the findings of this study are available from the corresponding author upon reasonable request.
